# Frailty predicts failure to discharge patients home from a subacute-care unit: a 3-year Italian experience

**DOI:** 10.1007/s40520-021-01908-w

**Published:** 2021-07-21

**Authors:** Paolo Mazzola, Valeria Buttò, Simona Elli, Riccardo Galluccio, Giulia Domenici, Valentina Stella, Justin Haas, Gianluca Peschi, Matteo Monzio Compagnoni, Giorgio Annoni, Giuseppe Bellelli

**Affiliations:** 1 School of Medicine and Surgery, University of Milano-Bicocca, U8 Building, Floor 4, Lab 4045 Via Cadore, 48, 20900 Monza, Italy; 2 Acute Geriatrics Unit, San Gerardo Hospital ASST Monza, Monza, Italy; 3 Department of Chronic Care (RICCA), Desio Hospital, Desio, Italy; 4 Clinical Neurosciences Research Area, NeuroMI, Milan Center for Neuroscience, Milano, Italy; 5 Faculty of Medicine, University of Toronto, Toronto, Canada; 6 Direzione Socio-Sanitaria, San Gerardo Hospital ASST Monza, Monza, Italy; 7 Unit of Biostatistics, Epidemiology and Public Health, Department of Statistics and Quantitative Methods, University of Milano-Bicocca, Milano, Italy; 8 National Centre for Healthcare Research and Pharmacoepidemiology, University of Milano-Bicocca, Milano, Italy

**Keywords:** Subacute care, Frailty, Elderly, Discharge home

## Abstract

**Background:**

The primary purpose of Subacute Care Units (SCU) is to decongest acute hospital wards and facilitate the return of older patients to home.

**Aims:**

We analyzed the clinical characteristics and outcomes of patients admitted to an Italian SCU, and we explored factors associated with discharge to locations other than home (outcome).

**Methods:**

This retrospective observational cohort study was conducted at a medium-sized suburban hospital, enrolling all patients consecutively admitted to one SCU from October 2017 to February 2020. We collected demographics, cause of admission, comorbidities, cognition, Barthel Index (BI), nutritional status, Clinical Frailty Scale (CFS), length of stay, and discharge destination. Delirium was screened with the 4AT score. We adopted a multivariable conditional logistic regression model to identify the factors associated with the outcome.

**Results:**

Frail subjects accounted for 58.6% of 406 patients (mean age 78.2 years, SD 11.6), while 61% were classified as functionally dependent. More than half of patients had relevant comorbidity, approximately 80% had a poor nutritional status, and 25% had pre-existing dementia. The overall prevalence of delirium was 14.5%. Most patients came from a hospital setting; recurrent reasons for admission were infections (70.5%) and heart failure (12.7%). Having a urinary bladder catheter at discharge, being overtly frail (CFS > 8), and low BI score were factors independently associated with not being discharged home.

**Discussion and conclusion:**

The routine assessment of frailty, as expressed by the CFS, may help redirecting the patients eligible for SCU admission.

## Introduction

Alternatives to hospital care for older patients are particularly important to consider in contexts where hospitals are overburdened (https://www.rcplondon.ac.uk/guidelines-policy/hospitals-edge-time-action) [1]. The introduction of intermediate care wards in the last few years has been proven as an innovative solution to the goal of preventing prolonged hospitalizations. These wards have been implemented as an intermediate step between the hospitalizations in acute wards and the return to home, a delicate phase requiring medical and paramedical interventions. Therefore, these wards are generally oriented to facilitate the transition more efficiently from the hospital to the home setting, and to promote patients’ functional independence. Thus, the objectives of the care provided in these wards are not primarily medical, but they are oriented towards the patient’s discharge destination and the achievement of a clinical outcome of recovery (or restoration of health) [2]. In Italy, the “Subacute Care Units” (SCUs) have been proposed as an interpretation of intermediate care units (Decree “*Delibera Regionale n. IX/1479 del 30/3/2011”*) [3]. To date, Lombardy is the most populated Italian region, with approximately 10 million residents accounting for nearly 16% of the total population of Italy [4]. In this regional framework, SCUs have so been implemented to relieve congestion on acute hospital wards and promote a comfortable and manageable return to home.

However, even though discharging patients home is one of the main objectives of the SCU system, there is little literature exploring the ability of these services in achieving this outcome.

In this study, we aim to report the clinical characteristics and outcomes of a cohort of patients residents in Lombardy region admitted to an SCU during a 3-year period. Since returning home after discharge is the principal objective of the SCU model of care, we also aim to identify the factors potentially associated with the lack of returning to home of these patients.

## Methods

### Setting

This study was conducted at a medium-sized suburban hospital located in Desio, a town of approximately 42,000 people, located in the Italian province of Monza-Brianza (850,000 inhabitants, http://demo.istat.it/index.html), Lombardy region.

The SCU of Desio Hospital opened on October 9th, 2017 as a 10-bed unit service managed by a team comprised of 1 physician acting as director, 2 geriatricians, 1 resident in geriatric medicine, 7 nurses, and other auxiliary personnel. Overall, nursing staff was assigned to work in such a way that each patient was allotted at least 180 min of direct nursing care per day.

### Regional criteria for the SCU admission

The criteria for admission to the SCU were established by the Regional Health Authority of Lombardy such that, to be transferred from acute hospital wards to the SCUs, a patient:

1. Should neither require intensive cardio-respiratory monitoring nor have drugs administered in continuous infusion;
2. Should not have “life-threatening” arrhythmias;
3. Should have independent breathing for at least 48 h (even if with oxygen support). The presence of a tracheotomy is not a contraindication;
4. Should neither have sepsis nor acute single or multi-organ failure;
5. Should not require a long-term use of nasogastric tube or percutaneous endoscopic gastrostomy;
6. Should not be on a waiting list for planned surgery;
7. Should give informed consent for admission to the SCU.

Conversely, to be admitted to an SCU from home:

1. The patient’s clinical status is such that he/she does not require admission to an acute hospital ward, but cannot be managed at home (for instance he/she requires low-tech diagnostic procedures or medical treatments that cannot be performed at home);
2. The patient needs clinical follow-up for recovery or stabilizing his/her health conditions.

### Study cohort

We enrolled all patients consecutively admitted to the SCU of Desio Hospital during the temporal period between October 2017 and February 2020. The date of the first admission to the SCU of Desio Hospital has been considered as the index date. There were no exclusion criteria, but, on admission, each patient (or his/her appointed substitute decision-maker, in case of the lack of capacity to understand) signed an informed consent form that allowed us to collect and use his/her medical information for academic publishing purposes. To preserve patient privacy, each identification code and all personal information were automatically anonymized in accordance with the Italian GDPR implementation law.

Data collection and all analyses were conducted according to the study research protocol (approved by the local Ethical Committee of ATS Brianza), adhering to the tenets of the Declaration of Helsinki of 1975 and its later amendments.

### Comprehensive geriatric assessment

For each patient, at the index date we collected demographic characteristics including age (in years), sex, living status), and reason for hospital admission, categorized as follows: infection, heart failure, recent surgery, metabolic, neurological, oncological, kidney disorders, and miscellaneous.

Comorbidity was assessed with the Charlson Comorbidity Index (CCI), a validated tool accounting for several chronic conditions that may affect 1-year survival [5]. Cognitive status was assessed by searching for a history of pre-existing dementia and using the mini-mental state examination (MMSE) [6]. We also collected information about the presence of delirium, which was assessed with the 4AT test [7].

Functional status was assessed using the Barthel Index (BI) [8] and by measuring the mobility level (able to walk with or without aids, bed-ridden). Nutritional status was evaluated with the mini nutritional assessment-short form (MNA-sf), a validated scale that is used to assign scores from 0 to 14 that estimate the risk of malnutrition (0–7: overt malnutrition; 8–11: at risk for malnutrition; 12–14: good nutritional status) [9].

The presence of frailty was described using the Clinical Frailty Scale (CFS), [10] a validated visual scale that subcategorizes the frailty phenotype. To date, the CFS reflects the baseline health status of a subject referring to 2 weeks before admission. For an accurate classification, it is crucial to identify subjects affected by a terminal condition (CFS 8 or 9). For those who are not, dependence on ADLs and IADLs helps to discriminate subjects characterized by higher levels of frailty (CFS 7 or 6) from subjects with mild frailty (CFS 5), respectively. If subjects are independent in ADLs and IADLs, chronic conditions and the perceived effort during daily activities help to identify very mildly frail individuals (CFS 4). Finally, doing sports or recreational activities at moderate-to-high intensity discriminates the individual’s level of fitness (CFS 1–3). For the purposes of this study, patients were assigned to one of four categories based on their CFS scores: fit (1 ≤ CFS ≤ 3), vulnerable or mildly frail (4 ≤ CFS ≤ 5), moderately to severely frail (6 ≤ CFS ≤ 7), and very severely frail or terminally ill (8 ≤ CFS ≤ 9).

We also collected data regarding the presence of pressure ulcers and a urinary bladder catheter, with reference to time before SCU admission and at discharge.

The assessment of CCI, cognitive status, presence of delirium, and of all the above-mentioned clinical characteristics was performed at the time of the first admission to the SCU (i.e., the index date) when data were collected during the anamnestic process.

Finally, we collected data regarding length of stay (LOS) in SCU and information on discharge destination, including home, non-home setting (e.g., hospital wards for new onset of acute conditions, or intermediate care, residential or rehabilitation facilities), in-hospital death or hospice.

### Statistical analysis

Continuous variables were described as mean ± standard deviation, or median and interquartile range where appropriate, whereas absolute frequencies and percentages were reported for categorical variables.

The admissions to SCU that did not end with a discharge home were labeled as outcome episodes and were considered as a proxy of a lack in the promotion of patients’ functional independence by the SCU. Patients were considered as not discharged home if they were discharged in a non-home setting like hospital wards for new acute conditions or intermediate care, residential or rehabilitation facilities, hospice, or if they died during the hospitalization. Comparisons among patients according to their discharge destination (i.e., home versus not home) were performed using (1) the Student’s *T* test for the means of two independent samples for continuous variables, and (2) the Chi-square test for categorical variables.

The stepwise selection method was applied in identifying and selecting factors able to predict the absence of a discharge home from the list of considered variables (see above), and a conditional logistic regression model was fitted to estimate the odds ratios (OR) and relative 95% confidence intervals (CI) for the association between those predisposing factors and the odds of being discharged to a location other than home.

Excel Software (from the Microsoft Office Personal Productivity Software Suite, Version 2019 16.0.6742.2048), the Statistical Analysis System Software (version 9·4; SAS Institute, Cary, NC, USA), and IBM SPSS software 24.0 Version (IBM Corp., Armonk, NY, USA) were used to perform all analyses. For all hypotheses tested, two-tailed *p* values less than 0.05 or, in an equivalent manner, 95% CI of Odds Ratio not containing the value expected under the null hypothesis were considered significant.

## Results

The first two columns of Table 1 show the demographic and clinical characteristics of our study population in total and according to the patient’s destination at discharge. We included 406 subjects, comparable in terms of sex (50.3% male and 49.7% female). The mean age was 78.2 (SD = 11.6) years, ranging from 19 to 103 years, with 127 patients (31.3%) aged 85 years and older. Most individuals lived with a family member (58.1%) or a caregiver (12.1%), and only eight patients (2%) lived in a nursing home before hospitalization in the SCU. Nearly nine out of ten patients admitted to the SCU came from an acute hospital setting, being medical wards a source of admission more common than surgical ones. The most common diagnosis at admission was infection (70.5%) and heart failure (12.7%), followed by a miscellaneous (8.4%), which included pulmonary conditions such as thromboembolism and various chronic respiratory diseases. The mean BI score was 48.8/100 (SD = 30.5), suggesting moderate-to-severe disability; only 7.9% of patients were bed-ridden, whereas 92.1% were able to walk with or without aids before hospitalization. The median CCI score was 3, and dementia accounted for 25.1% (102 patients). The mean MMSE score was 21.3 (SD = 7.5). According to the CFS, fit subjects accounted for 14.5% of the study population, vulnerable or mildly frail subjects for 26.9%, whereas almost half of the study cohort had a frailty degree ranging from moderate to severe, with 48% of patients scored with a CFS between 6 and 7. Patients with a CFS of 8 or greater accounted for 10.6% of the sample. According to the MNA-sf, one out of two participants were at risk for malnutrition, whereas 26.7% showed overt malnutrition. One in ten patients exhibited pre-existing pressure ulcers at admission to the SCU. Regarding urinary bladder catheterization, there was a reduction in its use from admission (29.3%) to discharge (12.6%). The overall prevalence of delirium was 14.5%. The average LOS was 16.5 (SD = 9.9) days. At discharge, most patients returned home (53.9%), while 187 subjects (42.9%) were discharged to a different setting. In-hospital death and access to hospice together accounted for 3.2% of the population. Destinations other than home included acute hospital wards (13.8%), intermediate care (23.2%), integrated assistance (35.4%), nursing home placement (16.6%), and rehabilitation facilities (3.9%).

**Table 1:** Baseline characteristics, demographics, and clinical features of patients admitted to the SCU at Desio Hospital, during the period between October 2017 and February 2020, according to discharge destination

Characteristics	Whole cohort (*N* = 406)	Discharged home (*N* = 217)	Not discharged home (*N* = 181)	*p *value
Sex				
Men	204 (50.3%)	110 (50.7%)	89 (49.2%)	0.7627
Women	202 (49.7%)	107 (49.3%)	92 (50.8%)	
Age (years)				
Mean (SD)	78.2 (11.6)	77.3 (12.2)	79.7 (10.7)	0.0405
18–64	50 (12.3%)	29 (13.4%)	19 (10.5%)	0.1311
65–74	64 (15.8%)	34 (15.7%)	27 (14.9%)	
75–84	165 (40.6%)	96 (44.2%)	67 (37.0%)	
≥ 85	127 (31.3%)	58 (26.7%)	68 (37.6%)	
Living arrangements^a^				
Alone	112 (27.8%)	64 (29.6%)	48 (26.8%)	0.0574
With a family member	234 (58.1%)	133 (61.6%)	101 (56.4%)	
With a caregiver	49 (12.1%)	19 (8.8%)	30 (16.8%)	
Nursing home	8 (2.0%)	–	–	
Source of admission^b^				
Acute medical hospital ward	359 (88.9%)	199 (92.1%)	156 (86.7%)	0.0982
Acute surgical hospital ward	30 (7.4%)	9 (4.2%)	19 (10.6%)	
Community-dwelling	12 (3.0%)	6 (2.8%)	4 (2.2%)	
Rehabilitation facilities	3 (0.7%)	2 (0.9%)	1 (0.6%)	
Diagnosis of admission ^a^				
Recent surgery	14 (3.5%)	1 (0.5%)	12 (6.6%)	0.0084
Infections	284 (70.5%)	156 (71.9%)	123 (68.0%)	
Congestive heart failure	51 (12.7%)	29 (13.4%)	22 (12.2%)	
Neurological diseases	4 (1.0%)	2 (0.9%)	2 (1.1%)	
Oncological diseases	4 (1.0%)	4 (1.8%)	0 (0%)	
Kidney diseases	2 (0.5%)	2 (0.9%)	0 (0%)	
Metabolic disorders	10 (2.5%)	7 (3.2%)	3 (1.7%)	
Miscellanea	34 (8.3%)	16 (7.4%)	19 (10.4%)	
Pre-hospital mobility				
Barthel index				
Mean (SD)	48.8 (30.5)	58.0 (30.1)	37.9 (27.1)	< 0.0001
Able to walk (with or without aids)	372 (92.1%)	206 (94.9%)	160 88.4(%)	0.0170
Bedridden	32 (7.9%)	10 (4.6%)	20 (11.1%)	0.0154
Charlson Comorbidity Index (CCI)				
Mean (SD)	3.0 (1.9)	3.0 (1.9)	2.9 (2.0)	0.6399
Dementia	98 (24.6%)	40 (18.4%)	58 (32.0%)	0.0017
Mini-Mental State Examination ^c^				
Mean (SD)	21.3 (7.5)	23.1 (6.3)	18.8 (8.3)	< 0.0001
Clinical frailty Scale ^d^				
Mean (SD)	5.6 (1.8)	4.9 (1.8)	6.4 (1.4)	< 0.0001
Fit (1–3)	59 (14.5%)	50 (23.0%)	8 (4.4%)	< 0.0001
Vulnerable or mild frail (4–5)	109 (26.9%)	76 (35.0%)	32 (17.7%)	
Moderate-to-severe frailty (6–7)	195 (48.0%)	81 (37.3%)	111 (61.3%)	
Very severe frailty or terminally ill (8–9)	43 (10.6%)	10 (4.7%)	30 (16.6%)	
Nutritional status ^f^				
Mini nutritional assessment				
Mean (SD)	8.9 (2.9)	9.5 (2.7)	8.2 (2.9)	< 0.0001
Malnourished	92 (26.7%)	34 (17.1%)	55 (39.0%)	< 0.0001
At risk	184 (53.5%)	113 (57.1%)	70 (49.7%)	
Well nourished	68 (19.8%)	51 (25.8%)	16 (11.3%)	
Pressure sores				
Pre-existing on admission	46 (11.3%)	12 (5.5%)	32 (17.7%)	< 0.0001
At discharge	50 (12.3%)	12 (5.5%)	36 (19.9%)	< 0.0001
Urinary bladder catheter				
On admission	119 (29.3%)	45 (20.7%)	71 (39.2%)	< 0.0001
Placed during SCU stay	71 (17.5%)	19 (8.8%)	51 (28.2%)	< 0.0001
At discharge	51 (12.6%)	8 (3.7%)	41 (22.7%)	< 0.0001
Delirium	59 (14.5%)	25 (11.6%)	33 (18.9%)	0.0460
Average length of stay in SCU (days)				
Mean (SD)	16.5 (9.9)	15.0 (7.9)	18.0 (11.6)	< 0.0001
Destination at discharge				
Home	219 (53.9%)	217 (100%)	0 (0%)	–
Non-home setting	174 (42.9%)	0 (0%)	168 (93.8%)	
Acute hospital ward	25 (13.8%)	–	25 (13.8%)	
Intermediate care	42 (23.2%)	–	42 (23.2%)	
Home care with multidisciplinary assistance	64 (35.4%)	–	64 (35.4%)	
Nursing home	30 (16.6%)	–	30 (16.6%)	
Rehabilitation facility	7 (3.9%)		7 (3.9%)	
In-hospital death or hospice	13 (3.2%)	0 (0%)	13 (7.2%)	

Italy, Desio, 2017–2020

^a^Calculated on 403 patients (395 for stratified analyses)

^**b**^Calculated on 404 patients (395 for stratified analyses)

^**c**^Calculated on 303 patients, because many subjects were not tested or not able to conduct the test (301 for stratified analyses)

^**d**^“*Fit*” includes Very fit, Well, Managing Well (CFS 1–3).“*Vulnerable or mild frail*” includes Vulnerable and Mildly Frail (CFS 4–5).“*Moderate to severe frailty*” includes Moderately Frail and Severely Frail (CFS 6–7).“*Very severe frailty or terminally ill*” includes CFS 8–9

^**e**^Calculated on 276 patients (275 for stratified analyses)

^**f**^Calculated on 344 patients (339 for stratified analyses)

Eight patients who already lived in nursing homes were excluded from the final analysis. The remaining 398 patients were categorized according to destination post-discharge (i.e., home versus not home) and their clinical characteristics are compared in Table 1.

Patients not discharged home were older than their counterparts (mean age of 79.7 years versus 77.0, *p* = 0.041). We also observed a different distribution (*p* = 0.057) for living arrangement prior to SCU admission: compared to those who were discharged home, patients not discharged home were more likely to have been living with a caregiver (16.8% vs. 8.8%), and less likely to have been living alone (26.8% vs. 29.6%) or with a family member (54.3% vs. 60.7%). Furthermore, patients discharged to a location other than home were mostly admitted to the SCU because of recent surgery (6.6% vs. 0.5%) or miscellaneous (10.4% vs. 7.4%), while infections and congestive heart failure were more common among patients discharged home (71.9% vs. 68.0%, and 13.4% vs. 12.2%, respectively). Patients not discharged home were also more disabled (mean BI 37.9 vs. 58.0, *p* < 0.0001), less able to walk (88.4% vs. 94.9%, *p* = 0.017), more commonly bed-ridden (11.1% vs. 4.6%) and more commonly affected by dementia (32.0% vs. 18.4%), as well as more frail according to the CFS score (mean score of 6.4 vs. 4.9, *p* < 0.0001). Furthermore, fit and vulnerable or mildly frail subjects prevailed among patients discharged home, while the frailest ones prevailed in the others. Patients not discharged home had poorer nutritional status (39.0% of patients were malnourished versus 17.1%), had more commonly pressure ulcers (both pre-existing on admission and at discharge), urinary bladder catheterization, and delirium than the ones discharged home. Finally, they also had a longer LOS (mean 18.0 days vs. 15.0, *p* < 0.0001) than others.

In a multivariable conditional logistic regression (Table 2), BI score, the presence of frailty at admission to the SCU, and of a urinary bladder catheter at discharge were associated with a discharge destination other than home. The lower the CFS score, the higher the likelihood of returning home. When considered as a continuous numeric variable, we found that each additional point of the CFS was associated with an increase of 60% in relative risk of being discharged to a destination other than home.

**Table 2:** Odds Ratios (OR), and 95% Confidence Intervals (CI), of not being discharged home after an admission to the Desio Hospital’s SCU, estimated by a multivariable conditional logistic regression model

Characteristics	OR (95% CI)
Diagnosis of admission^a^	
Miscellanea	Ref
Recent surgery	5.70 (0.61–49.5)
Infections	0.36 (0.15–0.82)
Congestive heart failure	0.27 (0.11–0.69)
Neurological diseases	0.14 (0.07–2.44)
Oncological diseases	NA
Kidney diseases	NA
Metabolic disorders	NA
Barthel index	0.98 (0.97–0.99)
Bedridden	1.82 (0.70–4.54)
Dementia	1.31 (0.72–2.41)
Clinical Frailty Scale^b^	
Not or very mild frailty (1–3)	Ref
Vulnerable or mild frail (4–5)	2.28 (0.92–5.57)
Moderate-to-severe frailty (6–7)	5.61 (2.20–14.48)
Very severe frailty or terminally ill (8–9)	6.41 (1.65–21.82)
Pressure sores	
At discharge	2.14 (0.95–4.81)
Urinary bladder catheter	
At discharge	5.34 (2.09–13.64)
Delirium on admission or during SCU stay	0.62 (0.31–1.24)

Italy, Desio, 2017–2020

^a^Calculated on 395 patients

^**b**^“*Not or very mild frailty*” includes Very fit, Well, Managing Well (RFS 1–3).“*Vulnerable or mild frail*” includes Vulnerable and Mildly Frail (RFS 4–5).“*Moderate to severe frailty*” includes Moderately Frail and Severely Frail (RFS 6–7).“*Very severe frailty or terminally ill*” includes RFS 8–9

## Discussion

In this study, we have described the clinical characteristics and outcomes of a cohort of patients admitted to our SCU during a 3-year period. We found that patients were old or very old and had significant burden in terms of disability and geriatric syndromes, including frailty and delirium. Around 46% of them did not return home after SCU stay, being transferred to different settings other than home and thus facing fragmentation of care. BI score, frailty levels, and the use of urinary catheter at discharge were the only factors independently associated with the risk of not being discharged home.

Our findings are similar to previous studies involving patients admitted to intermediate care units for heterogeneous reasons, i.e., not only for specific conditions such as those addressed to rehabilitation-oriented settings [11–16]. For example, we found that patients with impaired cognition, assessed with the MMSE (i.e., a score less than 24/30), accounted for 37.7% of the whole study population, a finding that is very similar to the one reported by Gual et al. [13] and Noaman et al. [14]. Delirium occurred in 14.5% of subjects, similar to the proportion reported by Bellelli et al. [11]. In the 2017 National Audit of Intermediate Care (NAIC) of England [17], a BI score at the admission of 54.1 for bed-based services (which include SCUs and nursing homes) was reported, a proportion in line with our findings. Some differences, probably due to distinct admission criteria across countries, were also observed between our study and previous studies also reporting on intermediate care units. For example, Sanchez-Rodriguez et al. reported lower BI values in their population [15]. Also, while we found that malnourished subjects accounted for 26.7% of our study population, with only 19.8% having a normal nutritional status, a study by Thomas et al. conducted in a subacute-care center in St. Louis reported an even higher percentage of subjects (about 90%) who were malnourished or at risk for malnutrition [16]. Overall, patients hospitalized in SCUs show a high level of complexity, as shown not only by comorbidities, but also by cognitive, functional status, nutritional status, and frailty.

An important finding of our study is that a frail phenotype in patients admitted to the SCU was independently associated with the risk of not being discharged home. We found that belonging to the “moderate to severe frailty” group and to the “very severe frailty or terminally ill” group increased the likelihood of being discharged to somewhere other than home by more than fivefold and sixfold, respectively. When modeling CFS on a continuous scale, the adjusted risk of not being discharged home increased with increasing frailty category, i.e., for each increase of one CFS point, the outcome risk increased by 60%.

We did not find any previous studies conducted in similar settings that used the CFS to assess frailty. In the study by Gual and colleagues, [23] patients were divided into two groups: chronic complex patients (CCP) and “unidentified”. The grouping criteria encompassed many features, including frailty, but the methods of assessment were not specified. Given the number of subjects in those groups (CCP = 91 and “unidentified” = 153), we could hypothesize that the prevalence of frailty is much lower than the one observed in our SCU, where frail subjects represent more than two-thirds of the study population.

To our knowledge, only one paper investigated the relationship between frailty and discharge destination in a setting like our own [18]. By administering the Edmonton Frail Scale (EFS) to patients aged 75 years and older, Haley and colleagues concluded that the routine assessment of frailty was not a useful predictor of destination post-discharge, dichotomized as “community (good outcome)” vs. “residential care (poor outcome)”. Instead, higher levels of frailty predicted a better level of participation in physiotherapy sessions [18]. However, due to missing data, the authors were only able to reach a final sample size of 75 subjects, which did not meet the number needed (*n* = 85) to detect a significant relationship between frailty and discharge outcomes (power 0.80, alpha 0.05). Moreover, the authors reported that the EFS might not have adequately captured the frailty phenotype in this cohort, considering that this scale seems to be more appropriate for frailty assessment among community-dwellers.

Our results have important implications: they show that CFS can be used to predict the discharge destination of patients admitted to an SCU and which variables indicate that a patient will likely be discharged somewhere other than home. The aim of SCUs is to avoid prolonged stays and to promote discharge to the community. SCUs therefore constitute a key element of the healthcare network, ideally providing the so-called “continuity of care” between hospital and community [19]. However, to derive efficacy from such a service, it is important to select patients appropriately. The finding that about half of our patients did not return home directly suggests that the current eligibility criteria provided by our Regional Healthcare System for admission to the SCUs should be revised. In fact, these criteria mainly consider clinical instability and the presence of acute diseases, but they do not consider frailty or other measures of individual biological reserve. Acute diseases and comorbidities are unidimensional measures of a patient’s health status, while frailty is a multidimensional measure, and therefore more appropriate to capture the complexity of an individual’s biological status [20]. We propose that the assessment of frailty should become a key element for eligibility to an SCU: our results clearly show that patients with higher levels of frailty do not benefit from SCU admission and that they re-enter the healthcare system with further periods of hospitalization across different settings of care. In turn, this “fragmentation of care” carries a further risk of physical and/or cognitive decline, which may lead to unfavorable outcomes, such as permanent institutionalization. Perhaps, subjects with increased levels of frailty should be redirected to more appropriate settings (such as rehabilitation-oriented settings, nursing homes, or hospice). Alternatively, national healthcare services should invest more resources in SCUs, e.g., funding the enrollment of necessary SCU personnel, such as physical therapists, occupational therapists, and experts in end-of-life care.

The finding that having a urinary bladder catheter at the end of the hospitalization increased the likelihood of not being discharged home is worth mentioning. Previous reports have already shown that urinary bladder catheterization is associated with poor health outcomes for the patient, including delirium, [21] urinary tract infections, sepsis, increased mortality, longer hospital stay, and higher care costs [22, 23]. Referring to the meta-analysis of Saint et al., about a quarter of patients with urinary bladder catheters develop bacteriuria. Of these, 24% evolve into symptoms of urinary tract infection and 3.6% into bacteremia [22]. Given that such conditions often require a high level of care, these patients are less likely to be discharged home. Since the use of urinary bladder catheterization is a modifiable risk factor, healthcare professionals should be aware of the implications of its prolonged use. Education about avoiding—or limiting wherever possible—the use of catheterization should be implemented throughout the levels of continuity of care.

The findings of this study should be interpreted considering its limitations. First, a single-center design limits the generalization of our findings to other care centers. Second, our SCU was opened physically adjacent to an acute Internal Medicine ward, and this fact could have indirectly affected the selection of patients. Indeed, we speculate that some patients with residual clinical instability may have been admitted to the SCU because of the proximity between the two wards. Moreover, variables describing the role of the caregiver and economic resources for each patient could have been relevant to interpret our outcome, as well as the reason for not being discharged home. However, these were not collected. A further limitation is represented by the sample size. Indeed, some of the variables tested in the logistic regression showed large confidence intervals, and therefore, we cannot exclude that, by increasing the study population, other variables may reach statistical significance in the multivariable analysis. Regarding the use of the CFS for frailty assessment, we acknowledge that this tool has not been validated to detect frailty in individuals younger than 65 years and we recognize this as a limitation of the study. However, it should be highlighted that frailty is common in younger adults too, especially if they undergo prolonged hospitalization, such as people admitted to the SCU. It is therefore likely that using the CFS, we have not overestimated the true proportion of frail individuals in our cohort. Finally, it is usual practice in our hospital that only a proportion of patients undergo rehabilitation. Indeed, given the shortage of the physical therapists, the selection of patients eligible for rehabilitation is based on a preliminary evaluation of the two physiatrists working in our hospital. Usually, they tend to select only the patients that they judge will benefit most from physiotherapy and it is very likely that only a minority of patients admitted to our SCU were among them. We recognize that this is a further limitation of our study.

The strengths of this study are manifold. First, participants were enrolled prospectively and consecutively, to minimize selection bias. Second, a comprehensive geriatric assessment was administered systematically to all hospitalized subjects. Third, the CFS and the comprehensive geriatric assessment were managed by experienced geriatricians, and nurses and the auxiliary personnel were trained in the use and administration of validated geriatric scales, with the aim to homogenize the evaluation method and data collection.

## Conclusions

This study suggests that a simple but informative assessment of frailty, such as the CFS, may help identify patients who will not be discharged home after SCU stay. Encompassing the CFS in the eligibility criteria for admission in the SCU, at least at a local level, may help to identify which patients are suitable for this setting.

## Data Availability

Data are stored at Desio Hospital, with access granted upon request to ASST Brianza Healthcare Management Unit.
